# Bundle sheath cell‐specific expression of chloroplast genes encoding subunits of the NADH dehydrogenase‐like complex in maize

**DOI:** 10.1111/tpj.70602

**Published:** 2025-12-14

**Authors:** Haruna Yano, Yuya Fukuta, Yoshiki Nihsimura, Toshiharu Shikanai

**Affiliations:** ^1^ Department of Botany, Graduate School of Science Kyoto University Kyoto 606‐8502 Japan; ^2^ Present address: Center for Advanced Biomedical Sciences (TWIns) Waseda University Tokyo 162‐0056 Japan

**Keywords:** chloroplast gene expression, C_4_ photosynthesis, NDH, PPR protein

## Abstract

C_4_ photosynthesis alleviates the limitation caused by the oxygenase activity of Rubisco by partitioning photosynthetic functions between two distinct cell types: bundle sheath cells (BSCs) and mesophyll cells (MCs). These cell types perform different steps of photosynthesis using specialized machinery, accompanied by differential expression of chloroplast genes. To uncover the underlying molecular mechanisms for this differentiation, we isolated BSCs and MCs and compared their chloroplast transcriptomes, focusing on the chloroplast NADH dehydrogenase‐like (NDH) complex, which is enriched in BSCs. To investigate whether RNA stabilization contributes to differential gene expression, we analyzed RNA footprints that reflect the binding of pentatricopeptide repeat (PPR) proteins to their RNA targets. We could not detect cell‐type‐specific accumulation of footprint RNAs. We then focused on transcriptional regulation, specifically on an operon that starts with the *rps15* gene. The operon includes six *ndh* genes and the *psaC* gene encoding a photosystem I subunit. Transcript levels of all genes in this operon were higher in BSCs than in MCs, suggesting coordinated regulation as a transcriptional unit. Based on the genomic location of the *rps15* gene within inverted repeats near the junctions on both sides of the small single copy region, we demonstrated that *rps15*, through two distinct promoters, is sufficient to drive preferential accumulation of downstream transcripts in BSCs.

## Significance Statement

The *rps15* operon in maize chloroplasts contains six *ndh* genes that are preferentially expressed in bundle sheath cells. The *rps15* gene, along with its two promoters, is sufficient to drive cell‐type‐specific accumulation of downstream transcripts. Both transcriptional control and RNA stabilization mechanisms likely contribute to this process.

## INTRODUCTION

Ribulose‐1,5‐bisphosphate carboxylase/oxygenase (Rubisco) catalyzes both the carboxylation and oxygenation of ribulose‐1,5‐bisphosphate, with the latter reaction producing 2‐phosphoglycolate, a byproduct that initiates photorespiration (Bauwe, 2023). To mitigate the detrimental effects of photorespiration on photosynthetic efficiency, C_4_ photosynthesis has independently evolved at least 66 times among angiosperms (Sage et al., 2012). In C_4_ plants, photosynthetic processes are spatially separated between two distinct cell types. In mesophyll cells (MCs), bicarbonate is initially fixed by phosphoenolpyruvate carboxylase, producing oxaloacetate, which is subsequently reduced to malate in NADP‐malic enzyme (ME)‐type C_4_ photosynthesis. The malate is then transported to bundle sheath cells (BSCs), where it is decarboxylated to pyruvate, thereby increasing the CO₂ concentration in the chloroplasts. This elevated CO₂ level promotes carboxylation by Rubisco while suppressing its oxygenation activity. However, regenerating phosphoenolpyruvate in MCs requires two additional ATP molecules per CO₂ fixed, making C_4_ photosynthesis more ATP‐demanding than C_3_ photosynthesis.

In NADP‐ME‐type C_4_ photosynthesis, reducing power, in addition to CO₂, is transferred from MCs to BSCs. The NADPH demand in BSCs is further modulated by the export of 3‐phosphoglycerate from BSCs to MCs (Mattinson & Kelly, 2025). As a result, the light reactions in BSCs are primarily required to generate ATP via cyclic electron transport around photosystem I (PSI). In angiosperms, PSI cyclic electron transport occurs through two distinct pathways: one that depends on the PROTON GRADIENT REGULATION 5 (PGR5) protein and the other that is mediated by the chloroplast NADH dehydrogenase‐like (NDH) complex (Yamori & Shikanai, 2016). In C_4_ photosynthesis, the NDH complex plays a major role in ATP production needed for the operation of the C_4_ cycle (Ogawa et al., 2023; Takabayashi et al., 2005; Ermakova et al., 2024). Reflecting this functional specialization, MCs preferentially accumulate photosystem II (PSII), while Rubisco and the NDH complex are more abundant in BSCs (Majeran et al., 2005). However, the mechanisms by which distinct chloroplast types with cell‐specific photosynthetic machinery are established remain poorly understood, particularly in terms of the regulation of chloroplast gene expression.

In angiosperms, the eleven core subunits of the chloroplast NDH complex are encoded by the chloroplast genome (Shikanai et al., 2025). Nuclear‐encoded factors essential for the expression of chloroplast *ndh* genes have been identified primarily through genetic studies in Arabidopsis (*Arabidopsis thaliana*). One such factor is CHLORORESPIRATORY REDUCTION 2 (CRR2), a member of the pentatricopeptide repeat (PPR) protein family, which recognizes RNA in a sequence‐specific manner and is involved in various steps of RNA maturation in plant chloroplasts and mitochondria (Barkan & Small, 2014). CRR2 is required for the formation or stabilization of *ndhB* mRNA with a specific 5' end (Hashimoto et al., 2003; Ruwe et al., 2019). The monocistronic RNA produced in a CRR2‐dependent manner is essential for *ndhB* translation. Accordingly, Arabidopsis mutants lacking CRR2 are unable to translate *ndhB*, resulting in the loss of the NDH complex (Hashimoto et al., 2003).

Despite this knowledge, it remains unclear how CRR2 contributes to the regulation of NDH complex abundance in response to physiological demands in wild‐type plants. In C_3_ plants such as Arabidopsis, the NDH complex accumulates broadly in green tissues and remains relatively stable once the thylakoid membrane is fully developed during early chloroplast development (Li et al., 2018). In contrast, in C_4_ plants, NDH assembly is expected to occur preferentially in BSCs. However, little is known about how chloroplast gene expression is regulated to achieve this cell‐type specificity in C_4_ plants. Based on the combination of RNA‐seq and ribo‐seq analysis, it was suggested that cell‐type‐specific protein accumulation is largely explained by the different accumulation of transcripts between MCs and BSCs in maize (*Zea mays*) (Chotewutmontri & Barkan, 2016). However, it is unclear whether this regulation occurs at the transcriptional or post‐transcriptional level through the control of RNA stability. In this study, we investigate the molecular mechanisms underlying cell‐type‐specific expression of chloroplast *ndh* genes in maize.

## RESULTS

### Cell‐type‐specific accumulation of transcripts in maize chloroplasts

**Figure 1:**
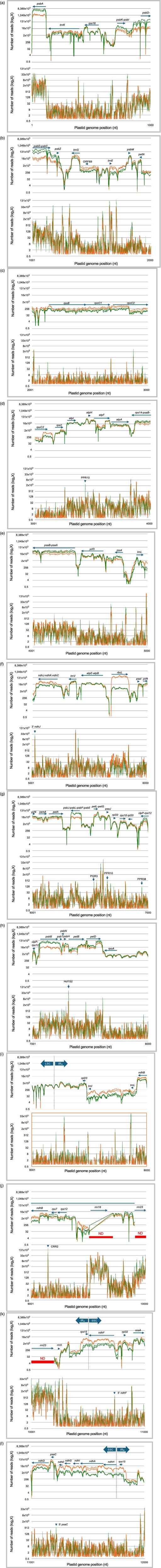
RNA‐seq reads are aligned across the maize chloroplast genome (X86563.2) (a‐l). Results for two reads are shown separately for each cell type: orange for BCSs and green for MCs. The number of reads for the long transcript is displayed in log_2_X in the upper panels. (b) The lower panels display the same analysis for small RNA, also plotted in log_2_X. The positions of representative genes are indicated. Junctions are marked among long single copy region (LSC), inverted repeat A (IR_A_), short single copy region (SCC), and IR_B_. ND; not determined because the regions encode rRNA genes.

To examine cell‐type‐specific transcript accumulation in maize chloroplasts, MCs and BSCs were independently isolated. MCs were separated from BSCs by protoplast preparation, while BSCs were isolated by mechanically disrupting MCs using a blender (see Methods). The purity of each cell fraction was verified by detecting marker proteins specifically localized to either MCs or BSCs (Figure S1). Total RNA was extracted from each cell type and subjected to RNA‐seq analysis. As shown in the upper panels (up) of Figure 1, individual transcript reads were aligned to the maize chloroplast genome. Transcripts derived from operons encoding PSII subunits, *psbA* and *psbK‐psbI* (Figure 1a, up), *psbD‐psbC‐psbZ* (Figure 1a,b, up), *psbE‐psbF‐psbL‐psbJ* (Figure 1g, up), and *psbB* and *psbT–psbH* (Figure 1h, up), were up to six times more abundant in MCs than in BSCs. In contrast, transcripts from the *rbcL* gene accumulated to higher levels in BSCs than in MCs, with a more pronounced difference than that observed for the PSII genes (Figure 1f, up). These transcriptomic patterns are consistent with previous reports (Kubicki et al., 1994; Sharpe et al., 2011; Chotewutmontri & Barkan, 2016) and the preferential accumulation of PSII and Rubisco in MCs and BSCs (Majeran et al., 2010), respectively.

Eleven subunit genes of the chloroplast NDH complex are organized into four distinct transcriptional units. The *ndhC*, *ndhK*, and *ndhJ* genes are located in the large single copy (LSC) region and are likely transcribed from a common promoter in that order. Transcript levels of this operon were approximately threefold higher in BSCs compared to MCs (Figure 1f, up). The *ndhB* gene is located within the inverted repeat (IR) regions and is presumably co‐transcribed with the upstream genes, *rps12* and *rps7* (Hashimoto et al., 2003). Transcript levels of *ndhB* were roughly twofold higher in BSCs than in MCs (Figure 1i,j, up). The remaining seven *ndh* genes are located in the small single copy (SSC) region. Among them, *ndhF* is transcribed as a monocistronic RNA, while the other six genes form an operon consisting of the *rps15*, *ndhH*, *ndhA*, *ndhI*, *ndhG*, *ndhE*, *psaC*, and *ndhD* genes in that order. Transcript levels for these genes were also consistently higher in BSCs than in MCs (Figure 1k,l, up). This pattern of cell‐type‐specific accumulation of *ndh* transcripts aligns with previously reported data from transcriptome analyses (Sharpe et al., 2011), ribosome profiling (Chotewutmontri & Barkan, 2016), and proteomic analyses (Majeran et al., 2010), which support preferential expression of NDH complex components in BSCs.

### Cell‐type‐specific accumulation of small RNAs

Chloroplast gene expression is extensively controlled through post‐transcriptional mechanisms (Kubicki et al., 1994). PPR proteins are central to this regulation: by binding to transcript termini, they both stabilize RNA and promote translation (Higashi et al., 2021; Prikryl et al., 2011). PPR binding sites are protected from endogenous exonucleases and appear as small RNA ‘footprints’ in deep‐sequencing data (Ruwe & Schmitz‐Linneweber, 2012; Zhelyazkova, Hammani, et al., 2012). To assess whether PPR‐mediated stabilization contributes to cell‐type specificity, we compared small RNA profiles from BSCs and MCs, as shown in the lower panels (low) of Figure 1. Because small RNAs can arise from multiple processes other than the binding of PPR proteins, interpreting their significance requires comparison with profiles from PPR‐deficient mutants. Therefore, we first examined known PPR‐binding sites to establish a baseline for further analyses.

PPR10 binds the intergenic regions between *atpI*‐*atpH* and *psaJ*‐*rpl33*, where it protects the 3′ ends of upstream genes and 5′ ends of downstream genes (Pfalz et al., 2009). Unexpectedly, small RNAs corresponding to the PPR10 footprint in the *atpI*‐*atpH* intergenic region were almost exclusively detected in MCs, with little observed in BSCs, even though the mRNA levels are similar in MCs and BSCs (Figure S2a). Because PPR10 loss abolishes chloroplast ATP synthase in the Arabidopsis mutants, its binding is required for photoautotrophic growth, yet the footprint is undetectable in BSCs. A weaker MC bias was seen in the *psaJ*‐*rpl33* intergenic footprint, which was 2–3 times higher in MCs despite equal transcript abundance in both cell types (Figure S2b). Footprints for PPR38 ortholog (at the *clpP*‐*rps12* junction) and HCF152 (at the *psbH*‐*petB* junction) also showed higher accumulation in MCs (Figure S2c,d). Likewise, PGR3 footprints at the *petL* 5′ UTR were more abundant in MCs, even though *petL* mRNA was modestly enriched in BSCs (Figure S2e). No PGR3 footprint was detectable at *ndhG* (Figure 1l, low).

CRR2, a PPR protein, binds the intergenic region between *rps7* and *ndhB* and is required for translation of *ndhB* mRNA (Hashimoto et al., 2003). Although *ndhB* transcripts accumulate more in BSCs than in MCs (Figure 1i,j, up), CRR2‐dependent RNA footprints at the *rps7‐ndhB* junction are more abundant in MCs (Figure S2f). This observation is particularly striking given that upstream *rps12* and *rps7*, which are co‐transcribed with *ndhB*, accumulate more in MCs, while the processed *ndhB* mRNA itself is enriched in BSCs (Figure 1i,j, up). The small RNA found in this region maps to the junction between the MC‐rich upstream (*rps7*) and the BSC‐rich downstream (*ndhB*). As a result, the read levels of long transcripts in this region are similar between the two cell types (Figure S2f). To explain the BSC‐specific enrichment of the downstream *ndhB* transcripts, one might predict enhanced stabilization of the processed *ndhB* mRNA in BSCs, potentially mediated by CRR2 binding near its 5' end. However, the greater abundance of CRR2‐dependent footprints in MCs argues against a simple model in which footprint density directly reflects stabilization activity. Compounding this puzzle, publicly available transcriptome data indicate that *CRR2* expression is higher in MCs than in BSCs (Maize eFP Browser, https://bar.utoronto.ca/transcriptomics/efp_maize/cgi‐bin/efpWeb.cgi?dataSource=maize_rice_comparison).

In the *ndhC‐ndhK‐ndhJ* operon, a prominent small‐RNA footprint maps to the 3′ UTR of *ndhJ* (Figure 1f, low), yet no known PPR protein has been assigned to this site. Like the CRR2‐associated footprints, this *ndhJ* small RNA is more abundant in MCs than in BSCs, even though long *ndhJ* transcripts accumulate to higher levels in BSCs (Figure S2g). Therefore, it is unlikely that a PPR protein binding at the *ndhJ* 3′ UTR mediates the BSC‐specific enrichment of transcripts in this operon. A small RNA footprint was also detected in the 5′ UTR of the *ndhF* gene. This small RNA is likely indicative of PPR protein binding since it exactly corresponds to the 5′ end of the processed mRNA (see below). However, this footprint is more abundant in MCs than in BSCs, despite *ndhF* long transcripts accumulating to a greater extent in BSCs (Figure S2h).

Since the two cell types were isolated using different procedures, differences in RNA recovery efficiency, particularly for small RNAs, may have affected the results. Although small RNAs generally appear more abundant in MCs than in BSCs, we cannot conclude that this reflects a biological difference, as it may be an artifact of differential extraction efficiency. A more reliable approach would be to qualitatively compare the ratio of small RNAs to their corresponding long transcripts across different genes. While a relatively higher ratio of small RNAs to long transcripts was observed in BSCs than in MCs in the *psaJ*‐*rpl33* region recognized by PPR10 and the *clpP*‐*rps12* region recognized by PPR38 (Figure S2b,c), the ratio was similar to or even smaller in BSCs for NDH‐related transcripts, even though the long transcripts for these genes were more abundant in BSCs than in MCs (Figure S2f‐h). The abundance of footprint RNA may depend on nuclease activity and the affinity of binding proteins for target RNA. We do not rule out the possibility that the binding of some PPR proteins regulates RNA stability in a cell‐type‐specific manner, although we were unable to obtain results supporting this idea.

### The *rps15* operon is transcribed from promoters located upstream of *rps15*

**Figure 2:**
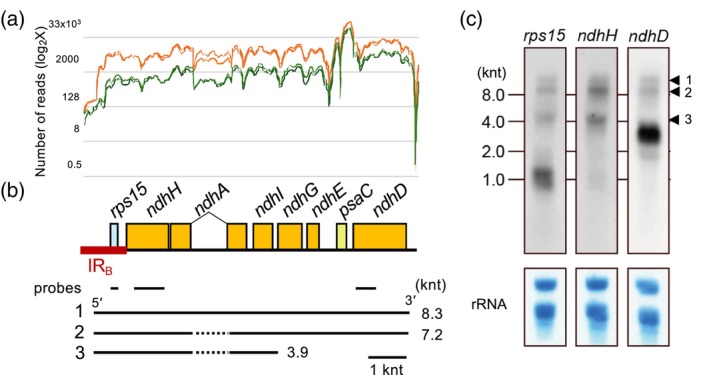
Analysis of transcripts in the *rps15* operon. (a) RNA‐seq reads are aligned across the chloroplast genome of the *rps15* operon, as shown in (b). The image is a close‐up of the corresponding region in Figure 1l, up. Results for two reads are shown separately for each cell type: orange for BCSs and green for MCs. The number of reads for the long transcript is displayed in log_2_X. (b) Map of the *rps15* operon. The positions of the probes used for RNA gel blot analysis are shown. The locations of three major transcripts are proposed. The position of the inverted repeat B (IR_B_) is marked. Since the *rps15* operon is encoded by the complementary strand in the genome data (X86563.2), it is shown as a mirror image in Figure 2 compared to Figures 1l and 3a. (c) RNA gel blot analysis of the *rps15*, *ndhH*, and *ndhD* genes. The positions of the molecular marker RNAs are indicated. Three arrows (1, 2, and 3) point to precursor or partially processed RNAs, with their positions proposed in (b). The rRNA bound to the membrane was stained with methylene blue.

It is possible that transcriptional regulation also contributes to the cell‐type‐specific expression of chloroplast genes in maize. To investigate this possibility, we focused on the operon that begins with the *rps15* gene, including six *ndh* genes and *psaC* (hereafter referred to as the *rps15* operon) (Figure 2b). The *rps15* gene encodes a component of the plastid small ribosomal subunit, whereas *psaC* encodes a stromal subunit of photosystem I (PSI). The six *ndh* genes in the operon encode subunits of the NDH complex, which mediates cyclic electron transport around PSI. The NDH‐dependent PSI cyclic electron transport is predominantly active in BSCs. Accordingly, their transcript levels were higher in BSCs than in MCs (Figure 2a). However, elevated expression of *rps15* in BSCs is not necessarily required, as its function is not specific to this cell type. Transcripts of *psaC* were also slightly more abundant in BSCs than in MCs. These observations suggest that transcript levels of genes within the same operon may be co‐regulated as a transcriptional unit, regardless of individual gene function or expression necessity in a specific cell type.

We analyzed transcripts originating from the *rps15* operon by RNA gel blot hybridization, using three probes specific to the *rps15*, *ndhH*, and *ndhD* genes, respectively (Figure 2b,c). In addition to shorter RNA species, likely representing processed transcripts, two longer RNA species were detected at approximately the 8.0‐knt position with all three probes. The pre‐stained size marker likely migrated slightly faster than the sample RNA due to its chemical properties. Based on their sizes, the longer transcript likely represents a primary precursor RNA encompassing all eight genes in the operon, while the shorter transcript may correspond to a spliced form from which the *ndhA* intron has been removed. The approximately 4‐kb band was also detected by two probes, *rps15* and *ndhH*. The signal probably corresponds to transcripts spanning from *rps15* to *ndhI*, with the intron of *ndhA* removed. These results indicate that the genes in the operon are transcribed from at least a promoter located upstream of the *rps15* gene.

Among the genes encoded by this operon, the transcript levels of the *ndh* genes generally accumulate to higher levels in BSCs, whereas the *psaC* transcript, which encodes a stromal subunit of PSI, is only mildly upregulated. This expression pattern likely underlies the upregulation of the NDH‐to‐PSI ratio in BSCs, which occurs in parallel at both the protein and transcript levels, presumably to meet the additional ATP demand of the C_4_ cycle (Takabayashi et al., 2005). While the NDH complex comprises only 1%–2% of PSI on a molar basis in C_3_ plants (Burrows et al., 1998), this ratio increases dramatically to approximately 50% in maize (Majeran et al., 2010). A comparable pattern was observed at the transcript level: in MCs, transcript levels of *psaC* were over twenty‐fold higher than those of the upstream *ndhE* gene, while in BSCs, the difference was narrowed to approximately ten‐fold (Figures 1l, up, and 2a).

To assess the mechanism underlying this distinct regulation of *psaC* transcript, we hypothesized that *psaC* might also be transcribed from its own promoter, in addition to the promoter upstream of *rps15*. In fact, the 5'‐end of RNA was detected in barley at the ‐188 position, which is likely protected by protein binding (Zhelyazkova, Hammani, et al., 2012). To test whether the end is generated by transcriptional initiation, we sought to distinguish primary transcripts from processed RNAs by analyzing their 5′ termini. In chloroplasts, primary transcripts possess 5′ triphosphate, which prevents adapter ligation in the 5′ RACE assay unless treated with Tobacco Acid Pyrophosphatase (TAP) to remove two terminal phosphates. In contrast, processed RNAs typically carry a 5′ monophosphate and can be ligated without TAP treatment. Thus, only processed transcripts are detected in the absence of TAP, whereas both primary and processed transcripts are detected after TAP treatment.

However, no difference was observed in the 5′ RACE products with and without TAP treatment, indicating that the 5′ ends of *psaC* transcripts arise from RNA processing rather than transcriptional initiation in maize (Figure S3a). To further characterize the 5′ termini, the major bands from TAP‐treated (band 1) and untreated samples (band 1′) were excised from the gel, cloned in *Escherichia coli*, and sequenced. The predominant 5′ end is located ~190 nts upstream of the *psaC* start codon, regardless of TAP treatment (Figure S3b), confirming that this site is generated via processing rather than transcriptional initiation. Notably, a peak of small RNAs overlapped this site (Figure 1l, low; Figures S2i, S3b), suggesting that RNA binding proteins may stabilize the processed 5′ end of the *psaC* mRNA.

We also considered the possibility that *psaC* might be transcribed from promoters located between *rps15* and *ndhE*. To test this, we performed 5′ RACE analyses to detect transcription initiation sites that are detected only after TAP treatment, using gene‐specific primers for *ndhI*, *ndhG*, *ndhE*, *ndhD*, and *ndhA* (including both exons and the intron) (Figure S3c). However, none of these primers yielded TAP‐dependent bands. These results suggest that the eight genes in the *rps15* operon are primarily transcribed from the promoter located upstream of *rps15* in maize.

### The *rps15* sequence, including its promoters, contributes to the BSC‐specific accumulation of the downstream transcripts

Transcript levels of all eight genes in the *rps15* operon are higher in BSCs than in MCs, although the increase is modest for *psaC* (~1.8‐fold; Figure 2a). This cell‐type specificity could arise either from elevated transcription of the precursor RNA in BSCs or from preferential protection of the precursor RNA from 5′ → 3′ exonuclease degradation in BSCs. In either case, the sequence encompassing the promoter and 5′ UTR of *rps15* (hereafter the 5′ region) would be sufficient to confer BSC‐specific transcript accumulation.

**Figure 3:**
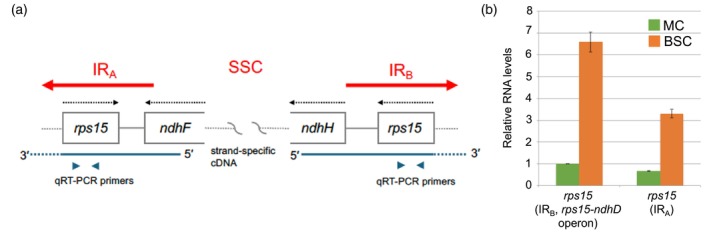
Contribution of the *rps15* gene to cell‐type‐specific expression of downstream genes. (a) Close‐ups of the junctions between inverted repeat A (IR_A_) and the small single copy region (SSC), and between SSC and IR_B_. The *rps15* gene present in IR_A_ is encoded by the opposite strand from the one encoding *ndhH*, *NdhF*, and *rps15* present in IR_B_. Positions of strand‐specific cDNA and primers used for qRT‐PCR are shown. (b) Relative RNA levels of transcripts from the *rps15* promoters in two cell types. The transcript level was normalized to the level of the *atpA* gene (*n* = 3, technical replicates). A similar result was obtained using independently isolated RNA samples.

In maize chloroplasts, the *rps15* gene and its 5' region reside in the inverted repeat B (IR_B_), whereas the downstream *ndhH* gene lies in the small single copy (SSC) region (Figure 3a). Although an identical *rps15* sequence is present in inverted repeat A (IR_A_), it is followed not by *ndhH*, but by the reverse complement of *ndhF*. We therefore interpreted the endogenous antisense *ndhF* transcript as a surrogate reporter gene to evaluate the contribution of the *rps15* 5' region to BSC‐specific expression of the operon.

To distinguish transcripts driven by the *rps15* promoter in IR_A_ versus IR_B_, we designed two primers for strand‐specific cDNA synthesis. A reverse primer in *ndhH* selectively primed cDNA synthesis of *rps15* transcripts originating from IR_B_, while a forward primer in *ndhF* targeted *rps15* transcripts from IR_A_ (Figure 3a). We then quantified *rps15* transcript levels by quantitative (q)RT‐PCR using each cDNA pool as a template. As an internal control, we co‐amplified *atpA*, which encodes a subunit of ATP synthase and exhibits uniform expression between BSCs and MCs (Figure 1d, up). Consistent with our RNA‐seq results (Figure 2a), *rps15* transcripts from IR_B_ were 6.6‐fold more abundant in BSCs than in MCs when normalized to *atpA* (Figure 3b). A similar BSC enrichment was observed for *rps15* transcripts from IR_A_, with 4.9‐fold higher abundance in BSCs. These data support that the *rps15* promoter and 5′ UTR region are capable of conferring BSC‐specific accumulation of downstream transcripts.

### Determination of the transcriptional initiation sites and the mature 5′ ends of *rps15* transcripts

**Figure 4:**
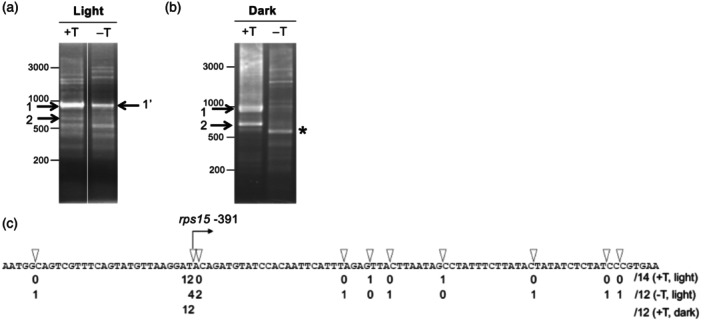
Determination of the 5′ ends of *rps15* mRNA. (a) Total RNA was ligated to the linker with Tobacco Acid Pyrophosphatase (TAP) treatment (+T) or without (−T). The resulting RT‐PCR products, using the infusion_ndhH_TAP_PCR primer (Table S1), were analyzed by agarose gel electrophoresis. (b) Total RNA was isolated from seedlings grown in the dark. RNA was ligated to the linker with TAP treatment (+T) or without (−T). The intensity of band 2 was higher in etiolated seedlings. The band corresponding to 1′ was absent, indicating that processed RNA was unstable in the dark. The identity of the band marked by an asterisk in the lane (−T) remains unclear. (c) The 5′ ends of *rps15* mRNA were identified after cloning band 1 and band 1′. The number of clones with each starting point is shown as a proportion of the total clones analyzed. The putative transcription start site (−391) is marked. The same analysis was performed after cloning band 1 of the dark‐grown seedlings (+T).

Since the *rps15* 5′ region drives BSC‐specific transcript accumulation (Figure 3), we mapped the transcriptional start site (TSS) and characterized the mature 5′ ends of *rps15* transcripts to study the mechanism for cell‐type‐specific expression. Because transcript levels are low in MCs, we could not exclude the possibility of BSC RNA contamination in MC preparation. Therefore, we performed 5′ RACE using total leaf RNA instead of attempting to analyze RNA isolated from each cell type. In 5′ RACE reactions, TAP treatment revealed a predominant band (band 1), whereas a corresponding but weaker band (band 1′) was also observed without TAP (Figure 4a). To determine whether band 1 represents primary transcripts and band 1′ corresponds to processed forms with truncated 5′ UTRs, we cloned and sequenced RACE products from both bands. Among 14 clones from the TAP‐treated sample, 12 mapped to position −391 relative to the *rps15* start codon (+1), identifying this site as the major TSS (Figure 4c). Notably, the GenBank reference (NC_001666.2) mis‐annotates the *rps15* translational start codon at the second AUG, located 36 nts downstream of the true site. Our mapping aligns with the −393 TSS reported in barley (Zhelyazkova, Sharm, et al., 2012). In contrast, the TAP‐untreated samples exhibited seven 5′ termini all within 65 nts downstream of position −391, indicating that primary transcripts are processed to yield a heterogeneous population of mature 5′ ends.

In our 5′ RACE assays, a second, weaker band (band 2) was detected only in the TAP‐treated sample (Figure 4a). Although we were unable to clone fragments from band 2, preventing precise identification of its TSS, the size of the RACE product suggests that this promoter is located approximately 200 nts downstream of the major −391 site. This position corresponds closely to the Trps15‐228 start site characterized in barley (Zhelyazkova, Hammani, et al., 2012; Zhelyazkova, Sharm, et al., 2012; Figure S4a). In barley, the Trps15‐228 promoter is recognized by the nuclear‐encoded plastid RNA polymerase (NEP) and is upregulated in etiolated seedlings. To test whether a similar regulatory mechanism operates in maize, we performed TAP assays on RNA extracted from dark‐grown seedlings. Consistent with NEP‐type activity, band 2 intensity increased markedly in etiolated samples (Figure 4b). In contrast, band 1′, representing the processed form of the major transcript, disappeared entirely, suggesting that processed RNA transcribed from the −391 promoter is unstable in the dark. Instead, a novel ~500‐bp band appeared in the untreated (–TAP) sample (asterisk in Figure 4b), possibly reflecting RNA protected by local secondary structure (Figure S4b). Although we could not find any RNA footprints in this region, the stability of RNA with the long 5′ UTR is regulated in response to light.

Together, these data indicate that the *rps15* operon is transcribed from two distinct promoters located upstream of *rps15*: a primary plastid‐encoded plastid RNA polymerase (PEP)‐dependent promoter at −391 and a secondary NEP‐dependent promoter around −230, the latter of which becomes more prominent under dark conditions.

### The sequence of the *rps15* 5′ UTR also contributes to forming the 3′ end of the *ndhF* transcripts in the complementary strand

In the 5′ UTR of *rps15*, nucleotides −208 to −158 (with +1 at the translational start) are predicted to form a large, stable stem‐loop structure (Figure S4b). Transcript coverage downstream of this hairpin is increased, indicating it helps stabilize RNA transcribed from the IR_B_ region (Figure 2a). However, primary transcripts initiated from the upstream −391 promoter are trimmed at variable positions upstream of this stem‐loop structure, indicating that most mature RNAs in light‐grown seedlings do not start precisely at the stem‐loop. Importantly, this same UTR sequence lies on the complementary strand that encodes *ndhF*. By forming a stable secondary structure, the stem‐loop likely acts as a physical barrier to 3′ → 5′ exonucleases, thereby defining the mature 3′ end of *ndhF* transcripts. Moreover, the alternative promoter (Trps15‐228) mapped 19‐nts upstream of the hairpin (based on barley data) would generate transcripts whose 5′ ends begin just upstream of the stem‐loop, allowing this structure to specifically stabilize transcripts from the downstream, NEP‐dependent promoter.

**Figure 5:**
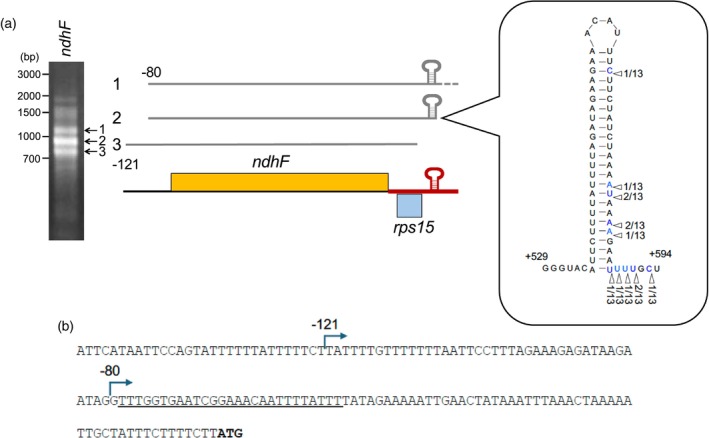
Determination of the 5′ and 3′ ends of *ndhF* transcripts. (a) Circular RT‐PCR identified two 5′ ends and three 3′ ends. The major transcript (band 2) ranges from −80 to near the end of the secondary structure. The number of clones with each 3′ end is shown as a proportion of the total. The band 1 transcript starts from the same −80 and has the 3′ extension across the secondary structure. The band 3 transcript starts upstream at −121 and is cut off in the 3′ secondary structure. (b) The sequence of the putative promoter and 5′ UTR region of the *ndhF* gene. An underline indicates a small RNA detected that matches the 5′ end (−80) of mature *ndhF* RNA (Figure 1k, low; Figure S2h), suggesting that a PPR protein protects the 5′ end of RNA.

The stem‐loop in the *rps15* 5′ UTR lies on the complementary strand that encodes *ndhF*, roughly corresponding to its mRNA 3′ terminus. To test whether this structure stabilizes *ndhF* transcripts, we mapped their 3′ ends by circular RT‐PCR, which simultaneously determines both 5′ and 3′ termini (Figure 5). Using the primer pair, we identified three distinct 3′‐end clusters, each associated with characteristic 5′ termini. The most abundant species (band 2) terminates immediately downstream of the predicted stem‐loop and carries a predominant 5′ terminus at −80 relative to the *ndhF* translational initiation site (Figure 5), indicating that the majority of *ndhF* mRNA utilizes the stem‐loop as their 3′ terminus. Longer products (band 1) extend beyond the hairpin, while band 3 species lack the stem‐loop entirely and carry a 5′ terminus at −121. Together, these data demonstrate that the stable secondary structure in the *rps15* 5′ UTR also functions to define and stabilize the 3′ termini of complementary *ndhF* transcripts. Therefore, when analyzing read coverage in the *rps15* 5′ region, it is necessary to consider transcripts from both DNA strands. On the sense strand, this region contains the −391 and −228 promoters driving the *rps15* operon; on the antisense strand, it overlaps the 3′ UTR of *ndhF*, spanning from the base of the predicted stem‐loop through the first nucleotide of *ndhH* (the IR_B_/SSC junction).

Because our RNA‐seq data are not strand‐specific, both sense and antisense transcripts contribute the read coverage. In BSCs, the initial rise in coverage starting at −391 can be attributed to transcription from the upstream *rps15* promoter in the IR_B_ region (Figure 2a). However, the second, sharper increase in reads at the stem‐loop region cannot be explained by the ‐391 promoter alone, since transcription occurs from both strands in this region. This increase therefore requires inclusion of *ndhF* transcripts initiated on the complementary strand, consistent with the observed upregulation of both *ndhF* and the *rps15* operon in BSCs relative to MCs (Figures 1k,l, up; 2a). The comparable read enrichment for *ndhF* and *rps15* indicates that the overall increase in local transcript abundance in BSCs reflects roughly equal contributions from the *rps15* 5' UTR and the complementary *ndhF* 3' UTR (as a transcript stabilization element).

## DISCUSSION

Our findings demonstrate that the sequence encompassing the *rps15* gene, including its promoters and 5' UTR, can confer BSC‐specific accumulation of downstream transcripts in the *rps15* operon (Figure 3). This cell‐type specificity may arise through two non‐mutually exclusive mechanisms: (1) enhanced transcriptional activity of the *rps15* operon in BSCs, and (2) preferential stabilization of the long precursor RNA in BSCs. Although we were unable to separate these two processes, we consider that both contribute to the regulation of cell‐type‐specific RNA accumulation.

We mapped two distinct TSSs upstream of *rps15*. The TSS at −391 precisely coincides with the barley Trps15‐393 site (Zhelyazkova, Sharm, et al., 2012) and is almost certainly recognized by PEP. The secondary TSS aligns with the NEP‐dependent Trps15‐228 promoter in barley. Its dark‐inducible behavior in maize seedlings (Figure 4b) further supports assignment to NEP. Thus, the *rps15* operon combines a PEP‐driven promoter, active in green tissues, with an NEP‐driven promoter that may assist the transcription in BSCs (see below). In Arabidopsis, sigma factor 4 (SIG4) directs PEP‐mediated transcription of *ndhF* (Favory et al., 2005), but this specificity factor is not conserved in monocots. Instead, maize SIG7 (Zm00001eb297490) is preferentially expressed in BSCs and represents a prime candidate for driving *ndhF* transcription in maize (Chotewutmontri & Barkan, 2021). SIG7 may also contribute to transcription from the *rps15*–391 site, thereby further reinforcing BSC‐specific expression.

Several canonical NEP‐transcribed genes, including *rpoC2*, *rpl32*, and various tRNA genes, exhibit higher transcript levels in BSCs than in MCs (Figure 1, up), suggesting that NEP activity is generally elevated in BSC chloroplasts. It is also possible that the downstream, NEP‐dependent *rps15* promoter contributes to the upregulation of *rps15* operon expression. The regulatory mechanism underlying this apparent upregulation of NEP activity, however, remains unclear as the gene encoding NEP does not show elevated expression in BSCs based on previously reported transcriptome data (Tausta et al., 2014).

Chloroplast gene expression is extensively regulated at the post‐transcriptional level, with PPR proteins playing crucial roles in this process (Small et al., 2023). Genetic studies have shown that PPR proteins are essential for proper gene expression by regulating RNA stability, maturation, RNA editing, and translational efficiency in a gene‐specific manner. To enable cell‐type‐specific chloroplast gene expression, it is plausible that the expression of PPR protein genes may also be modulated accordingly. As mentioned in the results section, careful consideration is necessary when interpreting the generally higher levels of RNA footprints seen in MCs compared to BSCs. This difference may result from varying efficiencies in small RNA preparation or, alternatively, reflect differences in nuclease activity between the two cell types. However, regardless of these possibilities, we could not find a clear correlation between the levels of RNA footprints and the corresponding long transcripts (Figure S2). For the conclusion, it would be necessary to directly analyze the cell‐type‐specific accumulation of PPR proteins.

Although we did not observe any clear PPR footprints within the 5′ region of *rps15*, we cannot rule out that PPR proteins contribute to its post‐transcriptional regulation. For example, PGR3 binds the 5′ UTR of *ndhG* to activate its translation, leaving a footprint in Arabidopsis (Higashi et al., 2021), but no corresponding footprints were detectable in our dataset in maize. Even in the absence of evidence for the involvement of PPR proteins, it remains reasonable to propose a mechanism in which the 5′ end of the *rps15* transcript is stabilized by the binding of a protein factor. The processed *rps15* transcripts, represented by band 1′, were absent in etiolated maize seedlings (Figure 4b), suggesting that in green tissue, the transcript is somehow protected from degradation. Protein factors other than PPR proteins may stabilize RNA independently of detectable RNA footprints (Bowman et al., 2013; Tillich et al., 2009). Alternatively, the RNA degradation machinery itself may exhibit selectivity, for example, by recognizing structural features of target RNA, such as secondary structure. In fact, the 5′ UTR of *rps15* and the 3′ UTR of *ndhF* share the same sequence in their complementary strands. By forming a secondary structure between the two RNA species, each RNA may help stabilize the other, as suggested in *Chlamydomonas reinhardtii* (Nishimura et al., 2004).

In Chlamydomonas, the translation of certain chloroplast‐encoded genes is attenuated in the absence of other subunits within the same photosynthetic complex (Choquet *et al*., 1998). This regulatory mechanism, known as control by epistasy of synthesis (CES), raises the possibility that a similar process could operate in maize to account for cell‐type‐specific patterns of transcript accumulation. However, CES may not be conserved to function this way, at least in the cases of ATP synthase in angiosperms (Zoschke et al., 2013). Recently, some CES‐like networks, not conserved in algal systems, were shown in tobacco, including ATP synthase (Ghandour et al., 2025). This work did not find the CES‐like regulation in the NDH complex in the Δ*ndhC*‐*ndhK*‐*ndhJ* and Δ*ndhA*‐*ndhI* mutants. In Arabidopsis, the *proton gradient regulation 3–1* (*pgr3‐1*) mutant fails to activate translation of the *ndhG* gene (Higashi et al., 2021). In this mutant, transcript levels of chloroplast‐encoded NDH subunit genes are mildly reduced, with the exception of *ndhB*. We do not rule out the possibility that the translation of NDH subunit genes and/or the stability of transcripts may be coordinated with the incorporation of their protein products into assembly intermediates in some subunits of the NDH complex.

In summary, our findings indicate that cell‐type‐specific gene expression in maize is largely determined by transcript abundance. This observation is consistent with previous studies, including ribosome profiling analyses of chloroplast translation (Chotewutmontri & Barkan, 2016). Changes in RNA levels during chloroplast development appear to primarily reflect differences in translational efficiency. We also found that transcript regulation generally occurs at the operon level. Although we cannot rule out the possibility, we did not find evidence supporting gene‐specific regulation of RNA stability through PPR protein binding in the context of cell‐type‐specific chloroplast gene expression. Rather than that, cell‐type‐specific RNA accumulation is likely regulated at the transcript level, with both transcriptional control and RNA stabilization at the transcriptional unit level playing a role.

## METHODS

### Plant materials and growth conditions

*Zea mays* B73 seedlings were cultured in soil at a photon flux density of 550 μmol photons m^−2^ s^−1^ (Figure 2) or 100 μmol photons m^−2^ s^−1^ under the 16‐h light (30°C)/8‐h dark (22°C) cycle. Tissues (9 cm above ligules) were collected as described in Li et al. (2010) from leaves 3–5 after 21 days.

### Isolation of cells

Cell separation was conducted following the protocol published in Takabayashi et al. (2005). For MC isolation, leaves were sliced into 1‐mm sections and treated with an enzyme solution containing 2% (w/v) cellulase (Onozuka R‐10, Yakult Pharmaceutical, Tokyo) and 0.5% (w/v) macerozyme (Onozuka R‐10, Yakult Pharmaceutical, Tokyo) in 0.5 M sorbitol, 5 mM MES‐KOH (pH 5.5), and 1 mM CaCl_2_ at 27°C for 4 h in the dark. After removing the enzyme solution, the tissues were washed with the same buffer without enzymes. The cell suspension was filtered through an 80‐μm nylon mesh, and cells were precipitated by centrifugation at 300*g* for 5 min at room temperature. The pellet was suspended in 0.5 M sucrose, 5 mM MES‐KOH (pH 6.5), and 1 mM CaCl_2_. The sorbitol solution was gently layered on the suspension and centrifuged again. MC was recovered from the interphase and washed with the sorbitol solution.

For the BSC isolation, leaves were sliced into 5‐mm sections and homogenized in buffer (0.3 M sorbitol, 50 mM MES‐KOH pH 6.1, 1 mM MgCl_2_, 1 mM MnCl_2_, 2 mM EDTA, 30 mM KCl, 0.25 mM KH_2_PO_4_) in a blender (Nissei DX‐5, Nihonseiki Kaisya, Tokyo) at 15000 rpm for 20 s. The suspension was filtered through an 80‐μm nylon mesh, and the residue was homogenized again. The procedure was repeated until the contaminating MC was completely removed, as confirmed by microscopic observation.

### RNA‐seq and small RNA‐seq

Total RNA was extracted from each cell lysate using the PureLinkTM RNA Mini Kit (Thermo Fisher Scientific, Waltham, MA, USA), and ribosomal RNA was removed with the Ribo‐ZeroTM Magnetic Kit (plant) (Illumina Inc., San Diego, CA, USA). The next‐generation sequencing (NGS) library was prepared using the TruSeq RNA Sample Prep Kit (Illumina Inc.), with individual barcode sequences added to each DNA fragment from different samples. These DNA fragments were pooled and subjected to double‐end sequencing on a HiSeq4000 (Illumina Inc.). Small RNA was isolated using the PureLinkTM miRNA Isolation Kit (Thermo Fisher Scientific). The NGS library was constructed utilizing the TruSeq small RNA Sample Prep Kit (Illumina Inc.), and the DNA fragments were subjected to single‐end sequencing on a HiSeq4000 (Illumina Inc.). Read counts per base in the chloroplast genome were normalized using the edgeR package in R. Normalization was performed with the TMM (Trimmed Mean of M‐values) method to account for differences in sequencing depth and library size across samples.

### RNA gel blot analysis

Total leaf RNA was extracted using the Maxwell 16 LEV Plant RNA Kit (Promega, Madison, WI). Five μg of RNA was loaded onto the denatured gel. After electrophoresis, the RNA was transferred to a Hybond N+ membrane (Cytiva, Amersham Pl., UK) and hybridized with a digoxigenin‐labeled DNA probe. DNA probes were prepared by PCR using primers listed in Table S1. The signals from the hybridized bands were detected using a Gene Image CDP‐Star Detection Kit (GE Healthcare, Piscataway, NJ).

### Quantitative RT‐PCR

To distinguish transcripts originating from the *rps15* promoter located in IR_A_ and IR_B_, strand‐specific cDNA was synthesized using primers, rps15‐IR_A_ (*ndhF*) and rps15‐IR_B_ (*ndhH*) (Figure 3a; Table S1). As an internal control, strand‐specific cDNA was also synthesized for the *atpA* gene using the primer atpA‐ssc. Quantitative (q)RT‐PCR was performed for *rps15* using primers, rps15‐F and rps15‐R, and for *atpA* using atpA‐F and atpA‐R. 500 ng of total RNA isolated from BSCs and MCs was used for cDNA synthesis. To prevent misannealing of strand‐specific primers, SuperScript III (Thermo Fisher Scientific) was used. FastStart Universal SYBR Green Master (Rox) (Roche, Basel, Switzerland) and MX3000P qPCR system were used for qRT‐PCR. Quantification was performed using the ΔΔCt (cycle threshold) method (Agilent Technologies, Santa Clara, CA).

### Determination of transcriptional start sites and processing of 5′ ends

The protocol was adapted from Swiatecka‐Hagenburch et al. (2007) and Kühn et al. (2005). Total RNA (1–5 μg) was treated with 0.5 U TAP (Invitrogen, Carlsbad, CA, USA) at 37°C for 1 h. After treatment with phenol:chloroform:isoamyl alcohol (25:24:1), RNA was recovered by ethanol precipitation. GeneRacerTM RNA origo (5′‐CGACUGGAGCACGAGGACACUGACAUGGACUGAAGGAGUAGAAA‐3′) (25 ng) was added, and the mixture was incubated at 65°C for 5 min, then transferred to ice. Next, the mixture was treated with 5 U T4 RNA ligase (Invitrogen, Carlsbad, CA, USA) at 37°C for 1 h, followed by RNA purification as described above. RNA was used for cDNA synthesis with 100 ng of random primer using SuperScriptTM III Reverse Transcriptase (Invitrogen). An aliquot was used for RT‐PCR with primers infusion_GeneRacer5_Fw and a gene‐specific reverse primer (Table S1) with 35 cycles. The product was separated on a 2% agarose gel, and the resulting band was excised. After In‐Fusion Cloning (Clontech, Palo Alto, CA, USA), the DNA was transformed into *Escherichia coli* DH5α. The plasmid was isolated and used for sequencing.

### Circular RT‐PCR

Both ends of RNA were ligated by T4 RNA ligase (Invitrogen) at 37°C for 1 h. After purification of RNA, the ligated RNA was used for RT‐PCR using random primers. cDNA was used for the second round of PCR using gene‐specific primers, cRT‐PCR_ndhF_Fw1 and cRT‐PCR_ndhF_Rv1 (Table S1).

## Author Contributions

All authors designed the research. HF conducted RNA‐seq analyses. HY examined the operons encoding the *ndh* genes. All authors reviewed the results and contributed to writing the manuscript.

## Conflict of Interest

The authors declare no conflicts of interest.

## Supporting information


**Figure S1.** Protein blot analysis of the marker protein for each cell type. A large subunit of Rubisco (RbcL) was detected as a marker for bundle sheath cells (BSC), whereas PEP carboxylase (PEPCsae) and PsbD (PSII subunit) were detected as markers for mesophyll cells (MC). Cytochrome *f* (Cyt*f*) is a subunit of the Cyt *b*_6_ *f* complex, which is present in both cell types. The positions of molecular size markers are indicated.
**Figure S2.** Quantitative analysis of footprint RNA. Number of reads for small RNA (footprints) and long RNA (transcripts), along with their ratio (small RNA/long RNA) are shown. Red and orange indicate two independent samples isolated from BSCs, while dark and light green represent samples from MCs. The *atpH‐atpI* (a) and *psaJ‐rpl33* (b) intergenic regions are recognized by PPR10. The *clpP‐rps12* (c) and *psbH‐petB* (d) intergenic regions are recognized by the PPR38 ortholog and Hcf152, respectively. PGR3 binds the 5′ UTR of *petL* (e), and CRR2 recognizes the *rps7‐ndhB* intergenic region (f). Peaks of small RNA were detected in the 3′ UTR of *ndhJ* (g) and the 5′ UTR of *ndhF* (h). The 5′ end of mature *psaC* mRNA aligns with the small RNA peak (i).
**Figure S3.** Determination of the 5′ ends of RNA. (a) Total RNA isolated from intact leaves was ligated to the linker with Tobacco Acid Pyrophosphatase (TAP) treatment (+T) or without (−T). The resulting RT‐PCR products were analyzed by agarose gel electrophoresis. Processed 5′ ends are visible in both lanes. Although the 5′ end, depending on transcription, should specifically appear in the +T lane, such a band was not observed. (b) The 5′ ends of RNA were mapped to the 5′ UTR of the *psaC* gene. The number of clones with each starting point is shown per total clones analyzed. The sequence corresponding to the peak of small RNA is highlighted in yellow. (c) Determination of the 5′ ends of RNA in the *ndhA*, *ndhI*, *ndhG*, *ndhE*, and *ndhD* genes. Total RNA was ligated to the linker with (+) or without (−) TAP treatment. RT‐PCR was performed using gene‐specific primers (Table S1). M, molecular marker.
**Figure S4.** Comparison of the promoter and 5′ UTR region of the *rps15* gene between maize (Zm) and barley (Hv). (a) Sequence alignment. The start codon of the *rps15* gene is marked in green. The plastid‐encoded plastid RNA polymerase (PEP)‐dependent upstream TSS (‐391 in maize) is indicated by arrows. The nuclear‐encoded plastid RNA polymerase (NEP)‐dependent TSS position (Trps15‐228) identified in barley is also shown. The maize sequence forming the stable secondary structure is highlighted in yellow, and the predicted secondary structure is shown (b). Underlines indicate the consensus sequences in barley for PEP and NEP recognition, respectively.

**Table S1.** Information of primers used in this study.

## Data Availability

The raw data for RNA‐seq is available in the DDBJ database under accession code, BioProject: PRJDB35920.
