# Supplementary material for: Bundle sheath cell‐specific expression of chloroplast genes encoding subunits of the NADH dehydrogenase‐like complex in maize
Source: Plant J. 2025 Dec 14;124(5):e70602. doi: 10.1111/tpj.70602 (PMC12702567; doi:10.1111/tpj.70602)
Supplement: Supplementary file 1 — Figure S1. Protein blot analysis of the marker protein for each cell type. A large subunit of Rubisco (RbcL) was detected as a marker for bundle sheath cells (BSC), whereas PEP carboxylase (PEPCsae) and PsbD (PSII subunit) were detected as markers for mesophyll cells (MC). Cytochrome f (Cytf) is a subunit of the Cyt b 6 f complex, which is present in both cell types. The positions of molecular size markers are indicated. Figure S2. Quantitative analysis of footprint RNA. Number of reads for small RNA (footprints) and long RNA (transcripts), along with their ratio (small RNA/long RNA) are shown. Red and orange indicate two independent samples isolated from BSCs, while dark and light green represent samples from MCs. The atpH‐atpI (a) and psaJ‐rpl33 (b) intergenic regions are recognized by PPR10. The clpP‐rps12 (c) and psbH‐petB (d) intergenic regions are recognized by the PPR38 ortholog and Hcf152, respectively. PGR3 binds the 5′ UTR of petL (e), and CRR2 recognizes the rps7‐ndhB intergenic region (f). Peaks of small RNA were detected in the 3′ UTR of ndhJ (g) and the 5′ UTR of ndhF (h). The 5′ end of mature psaC mRNA aligns with the small RNA peak (i). Figure S3. Determination of the 5′ ends of RNA. (a) Total RNA isolated from intact leaves was ligated to the linker with Tobacco Acid Pyrophosphatase (TAP) treatment (+T) or without (−T). The resulting RT‐PCR products were analyzed by agarose gel electrophoresis. Processed 5′ ends are visible in both lanes. Although the 5′ end, depending on transcription, should specifically appear in the +T lane, such a band was not observed. (b) The 5′ ends of RNA were mapped to the 5′ UTR of the psaC gene. The number of clones with each starting point is shown per total clones analyzed. The sequence corresponding to the peak of small RNA is highlighted in yellow. (c) Determination of the 5′ ends of RNA in the ndhA, ndhI, ndhG, ndhE, and ndhD genes. Total RNA was ligated to the linker with (+) or without (−) TAP treatment. RT‐PCR wa [file TPJ-124-0-s001.pptx]

## Slide 1
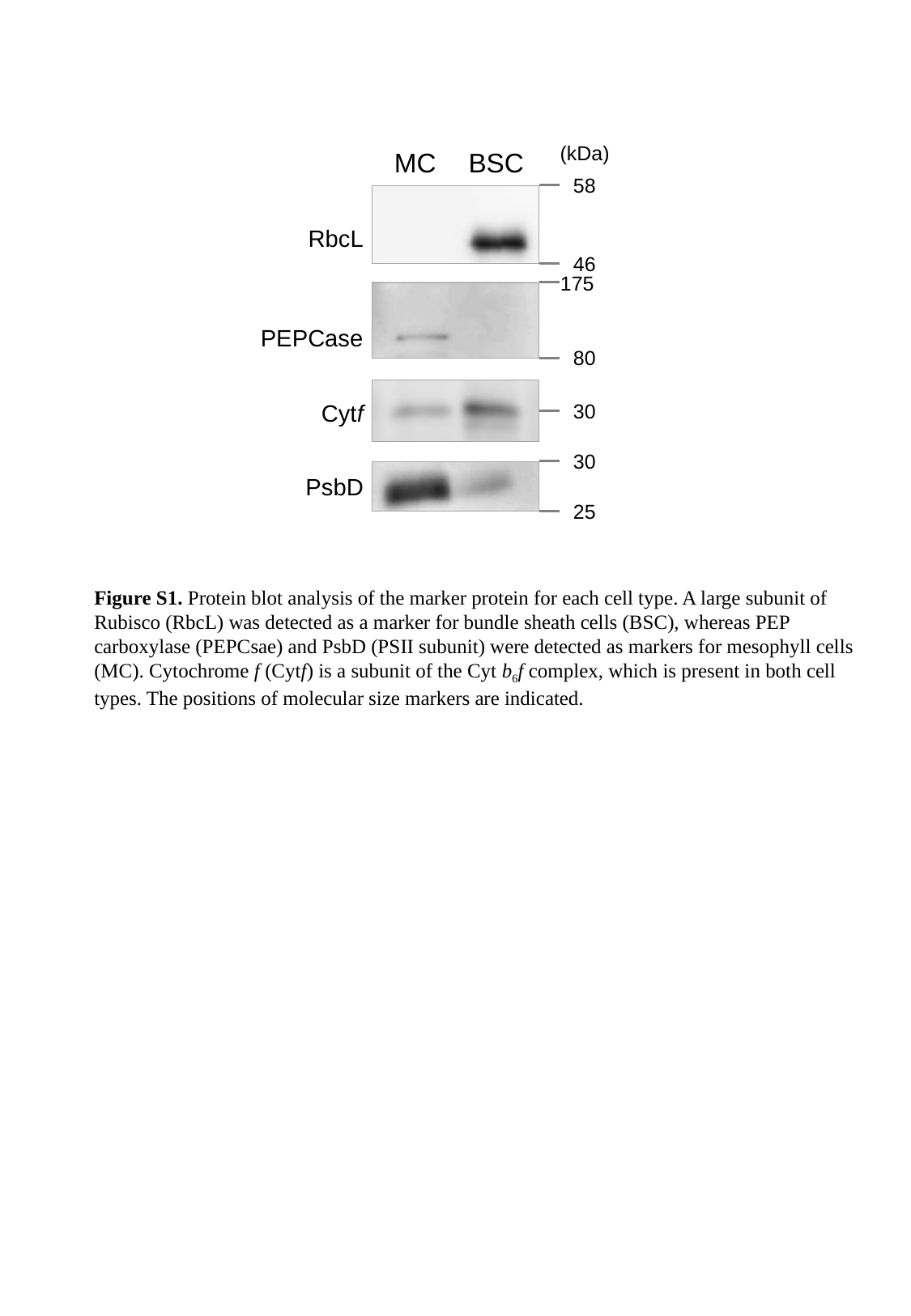

(kDa)
MC
BSC
58
RbcL
46
175
PEPCase
80
Cytf
30
30
PsbD
25
Figure S1. Protein blot analysis of the marker protein for each cell type. A large subunit of Rubisco (RbcL) was detected as a marker for bundle sheath cells (BSC), whereas PEP carboxylase (PEPCsae) and PsbD (PSII subunit) were detected as markers for mesophyll cells (MC). Cytochrome f (Cytf) is a subunit of the Cyt b6f complex, which is present in both cell types. The positions of molecular size markers are indicated.

## Slide 2
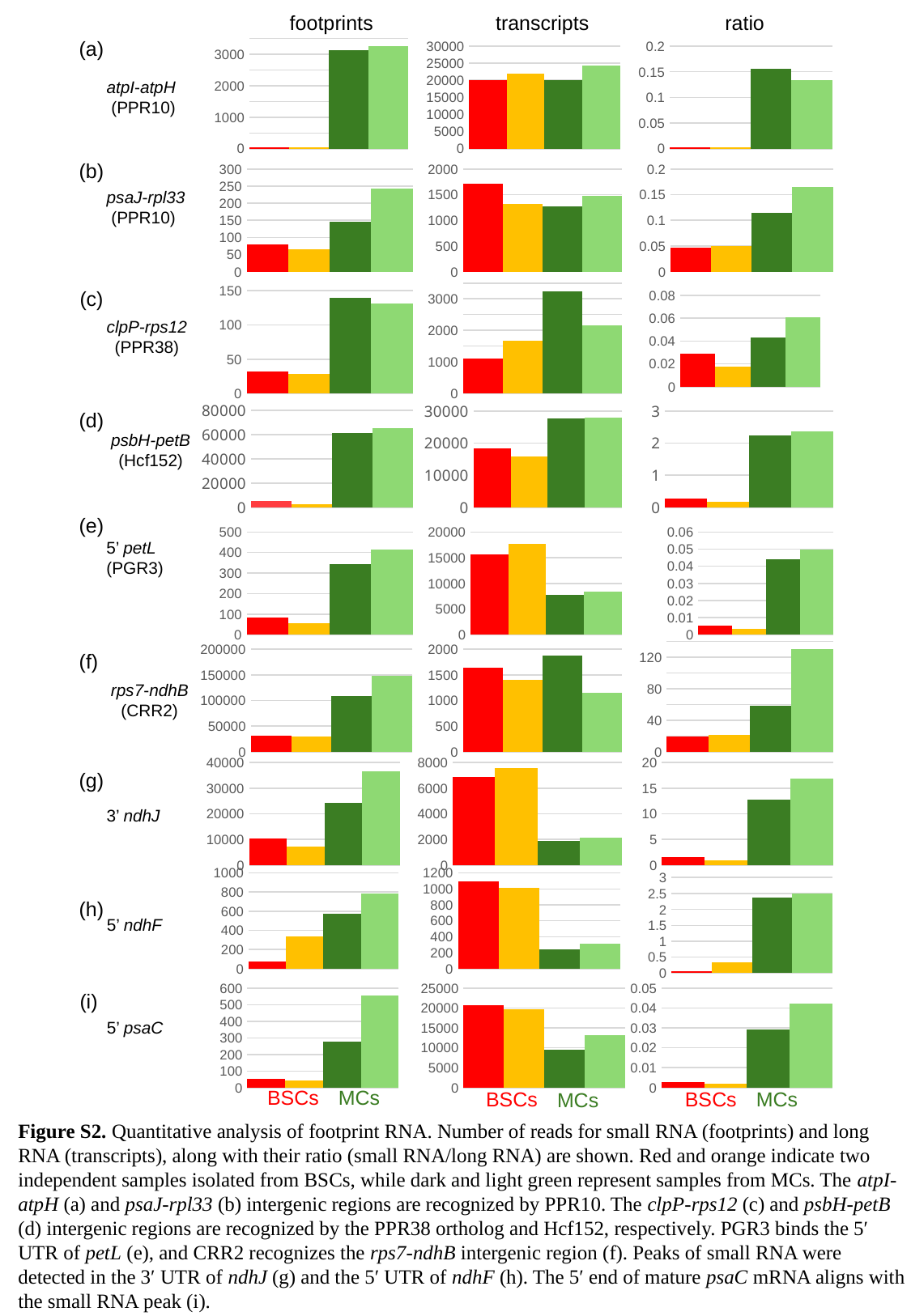

footprints
transcripts
ratio
(a)
### Chart
| Category | |
|---|---|
### Chart
| Category | |
|---|---|
### Chart
| Category | |
|---|---|
### Chart
| Category | |
|---|---|
### Chart
| Category | |
|---|---|
### Chart
| Category | |
|---|---|
### Chart
| Category | |
|---|---|
### Chart
| Category | |
|---|---|
### Chart
| Category | |
|---|---|
### Chart
| Category | |
|---|---|
### Chart
| Category | |
|---|---|
### Chart
| Category | |
|---|---|
### Chart
| Category | |
|---|---|
### Chart
| Category | |
|---|---|
### Chart
| Category | |
|---|---|
### Chart
| Category | |
|---|---|
### Chart
| Category | |
|---|---|
### Chart
| Category | |
|---|---|
### Chart
| Category | |
|---|---|
### Chart
| Category | |
|---|---|
### Chart
| Category | |
|---|---|
### Chart
| Category | |
|---|---|
### Chart
| Category | |
|---|---|
### Chart
| Category | |
|---|---|
### Chart
| Category | |
|---|---|
### Chart
| Category | |
|---|---|
### Chart
| Category | |
|---|---|atpI-atpH
 (PPR10)
(b)
psaJ-rpl33
 (PPR10)
(c)
clpP-rps12
(PPR38)
(d)
psbH-petB
(Hcf152)
(e)
5’ petL
(PGR3)
(f)
rps7-ndhB
(CRR2)
(g)
3’ ndhJ
(h)
5’ ndhF
(i)
5’ psaC
BSCs
MCs
BSCs
BSCs
MCs
MCs
Figure S2. Quantitative analysis of footprint RNA. Number of reads for small RNA (footprints) and long RNA (transcripts), along with their ratio (small RNA/long RNA) are shown. Red and orange indicate two independent samples isolated from BSCs, while dark and light green represent samples from MCs. The atpI-atpH (a) and psaJ-rpl33 (b) intergenic regions are recognized by PPR10. The clpP-rps12 (c) and psbH-petB (d) intergenic regions are recognized by the PPR38 ortholog and Hcf152, respectively. PGR3 binds the 5′ UTR of petL (e), and CRR2 recognizes the rps7-ndhB intergenic region (f). Peaks of small RNA were detected in the 3′ UTR of ndhJ (g) and the 5′ UTR of ndhF (h). The 5′ end of mature psaC mRNA aligns with the small RNA peak (i).

## Slide 3
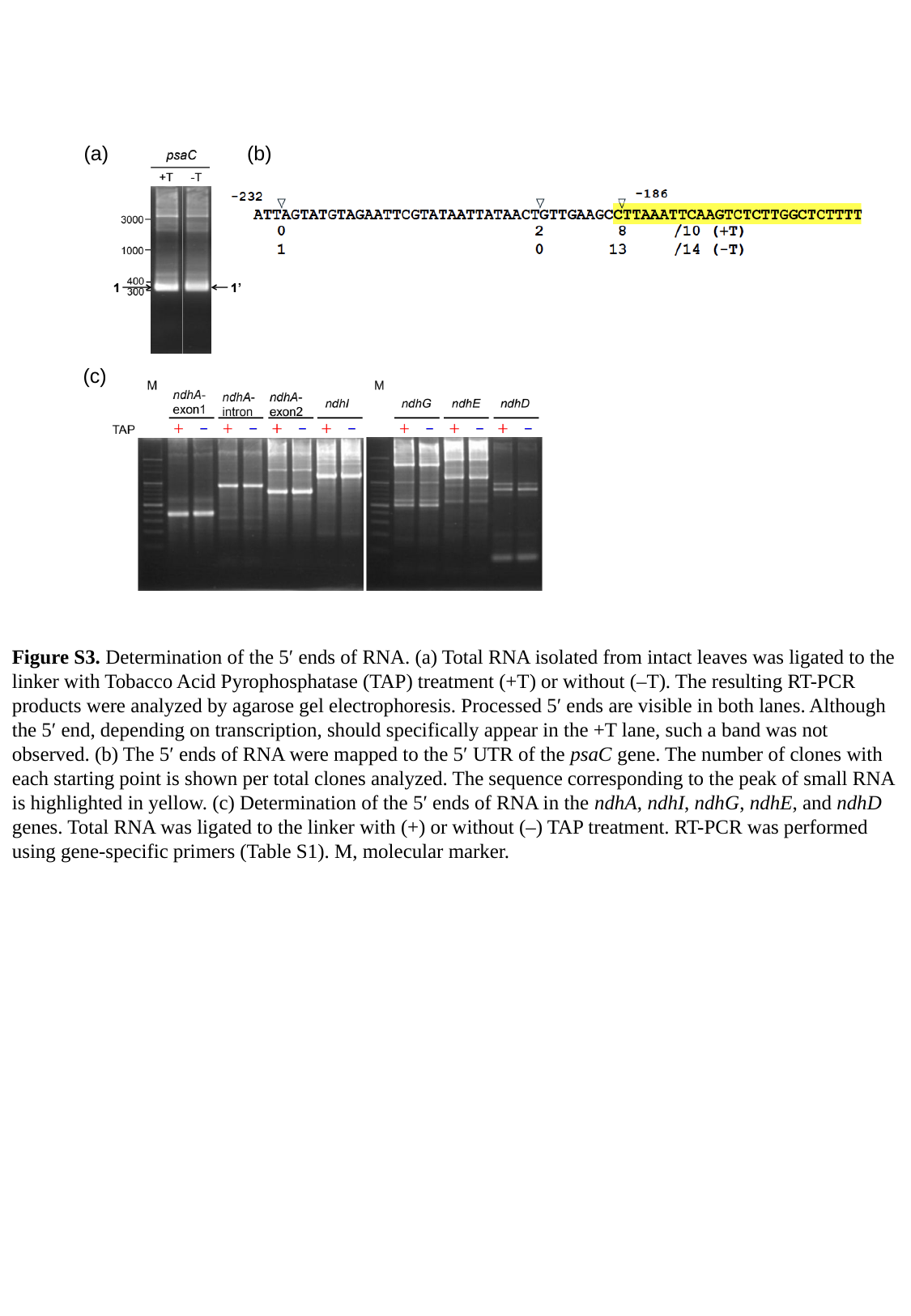

(a)
(b)
(c)
Figure S3. Determination of the 5′ ends of RNA. (a) Total RNA isolated from intact leaves was ligated to the linker with Tobacco Acid Pyrophosphatase (TAP) treatment (+T) or without (–T). The resulting RT-PCR products were analyzed by agarose gel electrophoresis. Processed 5′ ends are visible in both lanes. Although the 5′ end, depending on transcription, should specifically appear in the +T lane, such a band was not observed. (b) The 5′ ends of RNA were mapped to the 5′ UTR of the psaC gene. The number of clones with each starting point is shown per total clones analyzed. The sequence corresponding to the peak of small RNA is highlighted in yellow. (c) Determination of the 5′ ends of RNA in the ndhA, ndhI, ndhG, ndhE, and ndhD genes. Total RNA was ligated to the linker with (+) or without (–) TAP treatment. RT-PCR was performed using gene-specific primers (Table S1). M, molecular marker.

## Slide 4
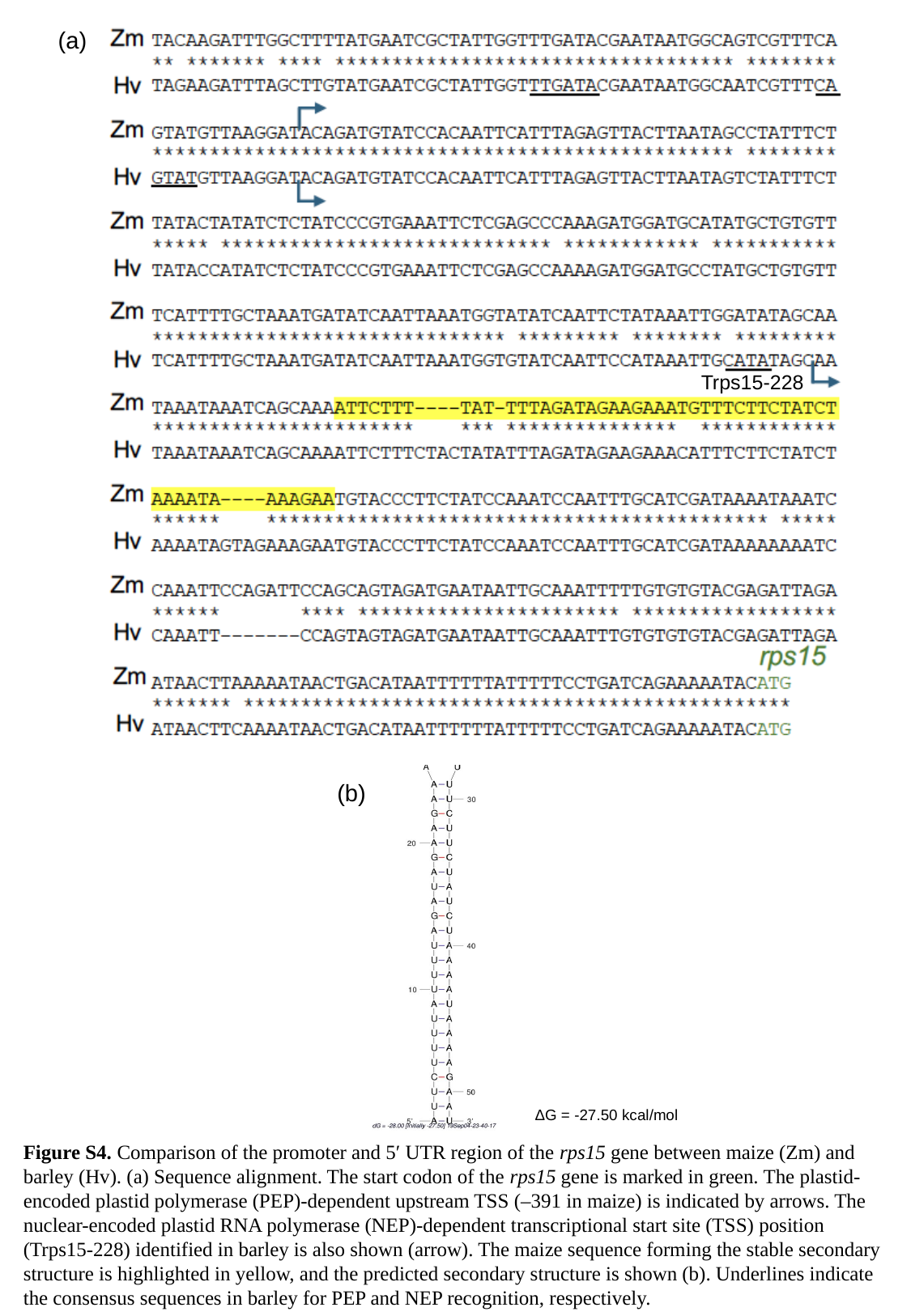

(a)
A
Trps15-228
(b)
ΔG = -27.50 kcal/mol
Figure S4. Comparison of the promoter and 5′ UTR region of the rps15 gene between maize (Zm) and barley (Hv). (a) Sequence alignment. The start codon of the rps15 gene is marked in green. The plastid-encoded plastid polymerase (PEP)-dependent upstream TSS (–391 in maize) is indicated by arrows. The nuclear-encoded plastid RNA polymerase (NEP)-dependent transcriptional start site (TSS) position (Trps15-228) identified in barley is also shown (arrow). The maize sequence forming the stable secondary structure is highlighted in yellow, and the predicted secondary structure is shown (b). Underlines indicate the consensus sequences in barley for PEP and NEP recognition, respectively.
